# Inequitable distribution of excess mortality during the COVID-19 pandemic in Korea, 2020

**DOI:** 10.4178/epih.e2022081

**Published:** 2022-09-26

**Authors:** Jin-Hwan Kim, Saerom Kim, Eunhye Park, Chang-yup Kim

**Affiliations:** 1 Department of Public Health, Graduate School of Public Health, Seoul National University, Seoul, Korea; 2 People’s Health Institute, Seoul, Korea

**Keywords:** COVID-19, Excess mortality, Equity, Life expectancy, Republic of Korea

## Abstract

**OBJECTIVES:**

This study analyzed inequities in excess mortality according to region and socioeconomic position to explain the distribution of excess mortality in Korea in 2020.

**METHODS:**

We acquired weekly all-cause mortality data from January 2015 to December 2020 from (1) the National Health Insurance Database and (2) Vital Statistics. Excess mortality for 2020 was calculated by comparing the weekly observed and expected deaths from the same period (2015-2019) using quasi-Poisson regression.

**RESULTS:**

An inequitable distribution of excess mortality was identified. The estimated excess mortality in Korea was -29,112 (95% confidence interval, -29,832 to -28,391), corresponding to -55 per 100,000, and the ratio of observed deaths to expected deaths was 0.91. Negative excess mortality was observed except for females in the 0-14 age group. Male Medical Aid beneficiaries showed positive excess mortality, while non-disabled and disabled groups showed similar negative values. When the standardized mortality ratio was calculated for the top 10 causes of death, deaths from Alzheimer’s disease and septicemia increased, whereas those from diabetes mellitus and cerebrovascular disease decreased. The decrease in mortality was primarily concentrated in older adults, while the mortality of young females increased due to increased intentional self-harm.

**CONCLUSIONS:**

This study adds essential evidence regarding the overall performance of Korea. The observed inequalities according to various socioeconomic variables indicate that the results of strict measures to control coronavirus disease 2019 were not distributed equitably. Efforts should be made to properly evaluate the current and future problems related to the pandemic.

## INTRODUCTION

Excess mortality has received much attention since the coronavirus disease 2019 (COVID-19) pandemic began due to the potential of this indicator to reflect complex outcomes of interactions between pandemic-induced changes in the mortality structure and adaptations to social policies [1,2]. Excess mortality is considered an indicator of unidentified COVID-19-related deaths, especially when testing capacities are insufficient [3-6]. However, in Korea, where the scale of the pandemic has been relatively small, and an elimination strategy was chosen [7], excess mortality is considered as an indicator of the overall change in the mortality structure related to the COVID-19 pandemic and policies to counteract it (Supplementary Material 1).

Despite the importance of excess mortality as a proxy parameter for determining the scale of a disaster [8] and the need for weekly reporting of excess mortality [9], excess mortality in Korea has not been fully explored. An international comparison study, which reported excess mortality in 29 high-income countries using standardized methodology, estimated 4,000 (2,900-5,000) excess deaths in Korea in 2020. However, this result was based on predicted deaths in 2020 based on data from previous years [10]. Another international study, which analyzed 40 high-income countries, reported that Korea had 560 (95% credible interval, -10,870 to 11,000) excess deaths from mid-February 2020 through mid-February 2021. That study used a Poisson-based regression model, but divided age into 3 groups (0-44, 45-64, and 65+ years), but only 2 groups (0-64 and 65+ years) in Korea. It also modeled the mortality structure, including trend adjustment, autocorrelation between years, potential influence from temperature, and the holiday effect [11].

Other estimates from Korean studies are also available from governmental reports. Statistics Korea provided the excess mortality profile by comparing the deaths reported over the last 3 years [12]. The preliminary results from the Health Insurance Review and Assessment Service of Korea reported that observed deaths were within the range of expected mortality during the COVID-19 pandemic period [13]. A study using vital statistics also identified decreases in overall mortality. That study found that there was a decrease in deaths from diseases of the respiratory system, while out-of-hospital deaths from neoplasms, diseases of the digestive system, non-accidental causes, and diseases of the circulatory system increased [14]. Another study using vital statistics and monthly suicidal death statistics from Statistics Korea identified female’s excess mortality due to self-harm in January and March 2021 [15].

However, the previous studies were insufficient in 2 aspects. First, estimates from granular data sources using methodologies comparable to those of other country-specific studies have not been reported. Second, knowledge of the unequal distribution of excess mortality has not been generated. We need to explore the unequal distribution of excess mortality, which can be masked in analyses of the entire population as a whole due to bi-directional changes between subpopulations. Despite several reports of health inequalities during the COVID-19 pandemic [16-19], research on the unequal distribution of COVID-19 pandemic-related mortality is especially sparse.

To explore the inequalities in this pandemic and their impacts, it is necessary to calculate a comparable estimate of excess mortality using previously validated methods and to explore the unequal distribution of deaths by considering various demographic factors, such as age, gender, region, income level, health coverage status, and disability. The primary objective of this study was to compute the magnitude of excess mortality and to analyze variations in excess mortality according to demographic and socioeconomic strata. We calculated estimates of excess mortality in Korea to identify the inequitable distribution of excess mortality according to various socioeconomic variables. The second purpose of the study was to explore the causes of the observed excess mortality. We calculated the standardized mortality rates (SMRs) of the top 10 causes of death and estimated the extent of each cause contributing to the change in life expectancy.

## MATERIALS AND METHODS

### Data collection

We utilized 2 mortality data from 2 sources, the National Health Insurance Database (NHID) provided by the National Health Insurance Service (NHIS) (https://nhiss.nhis.or.kr), and the vital statistics produced by Statistics Korea (https://mdis.kostat.go.kr). The NHID collects data on demographic variables (e.g., age, sex, insurance contribution, disability status) and other variables related to socioeconomic position [20]. The NHIS provided aggregated weekly death records by age group, sex, region, insurance contribution, and disability status from January 1, 2011 to December 31, 2020. Each year, there were 53 weekly reports. The data from the 53rd week were discarded from the analysis as they included only 1 day or 2 days of data, depending on whether it was a leap year, which resulted in excessive zeros when disaggregated (Supplementary Material 2). The age groups were classified according to 5-year intervals (0-4, 5-9, 10-14, 15-19, 20-24, 25-29, 30-34, 35-39, 40-44, 45-49, 50-54, 55-59, 60-64, 65-69, 70-74, 75-79, 80-84, and ≥ 85 years), and then regrouped according to the classification in previous studies for comparison. Regions were divided into 17 administrative provinces. Insurance contribution was used as a proxy indicator of income and was divided into 5 groups (0: Medical Aid beneficiary, 1-4: income quartiles 1 to 4 among health insurance enrollees). Individuals were classified by disability status as non-disabled if their disability rating code was “00” (normal), and others as disabled.

The raw data used in this study are not publicly shared and are only accessible when the NHIS authorizes anonymized utilization of the data for use in policy and research. Vital statistics were obtained using information from both the NHID and Statistics Korea. Although the death records in the 2 sources are not completely consistent, they can be considered as almost the same data at the population level [21]. Lastly, we assumed that there would be no significant delay in death reporting, considering the time when the data were collected and the accuracy of the vital statistics system in Korea.

### Statistical analysis

We calculated the expected number of deaths using a quasiPoisson regression analysis to deal with overdispersion. We modeled weekly deaths from 2015 to 2019 as a Poisson-distributed random variable with possible overdispersion. Although it has been proposed that the mortality pattern in the winter of 2018 was an extension of an existing trend [22], we performed a sensitivity analysis by calculating estimates that excluded the cold-wave period (week 49 in 2017 to week 18 in 2018, hereafter referred to as the cold-wave-adjusted estimate) [10].


Deathi,j,k,l=populationi,k,k,lexp(β0+β1,i⋅Weeki+β2,j⋅Sexj+β3,k⋅Age groupk+β4,j,k⋅SEXj⋅Age groupk+β5,l⋅Regionl

where *Death_i, j, k, l_* is the expected number of deaths in week *i*, sex *k*, age group *j*, and region *l*.

We used a generalized linear model with the *glm* function of the quasi-Poisson family in R (R Core Team, Vienna, Austria). The number of expected deaths was calculated for each combination, and the results were shown as aggregates. The confidence interval (CI) was computed using the block bootstrap method, which resampled the data with replacement in each combination (week, age group, sex, and region) and provided the number of deaths that would have occurred in the absence of the COVID-19 pandemic in 2020. CIs for each region were obtained by calculating the 2.5th and 97.5th percentiles of the rolled-up bootstrap estimates, which were subsequently compared with the results obtained by using vital statistics from Statistics Korea in the same model to check their validity. The demographic data provided by the Ministry of the Interior and Safety were used as denominators, as the vital statistics were determined for all Koreans, whereas the NHID was used for those with health insurance regardless of nationality. Next, we created additional models that included income quintiles, disability status, and health coverage type as covariates and calculated rates per 100,000 to correct the difference in subpopulation size and the ratio of observed and expected deaths to capture the possibility of excess mortality in opposite directions. Furthermore, each indicator measures the absolute and relative dimensions of health inequalities [23,24].

Finally, 2 additional analyses were performed to understand the changes in the mortality structure. Indirect standardization was conducted for age-specific and sex-specific rates from 2015 to 2019 for the top 10 causes of death to identify the diseases from which excess mortality occurred. With due consideration of the stability of the analysis, we used the classification of 56 causes of death provided by Statistics Korea, instead of the 103 causes of death used by the World Health Organization (Supplementary Material 3). Decomposition of life expectancy, using a stepwise replacement algorithm provided by DemoDecomp of the R package [25], was performed to understand the absolute contribution of each cause of death. Decomposition was conducted by comparing statistics of 2015-2019 and 2020 similarly to the other analyses. All statistical analyses were performed in R version 4.1.2 (R Core Team).

### Ethics statement

The Institutional Review Board of Seoul National University waived the requirement of ethics review for this research as the study used anonymized data (IRB No. E2106-001-005 and E2109/001-002).

## RESULTS

### Excess mortality at the national level

The overall death toll in Korea was 274,848, 278,870, 284,466, 297,495, and 293,937 in 2015, 2016, 2017, 2018, and 2019, respectively. The death toll reached 303,014 in 2020. As the cold wave in the winter of 2018 caused many deaths, the death toll increased in 2018, but subsequently decreased in 2019. Thus, in 2020, only mortality in week 18 (April 29 to March 5) and weeks 33-35 (August 12 to September 1) seemed to deviate from the trends of the previous years (2015-2019) (Supplementary Material 1).

**Figure 1.**
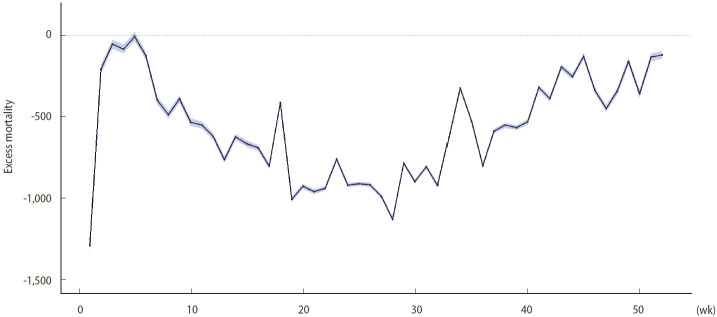
Weekly excess mortality in 2020. The *x*-axis represents the number of weeks, and the *y*-axis represents the estimated excess mortality per week. The bootstrapped 95% confidence interval is indicated by a blue-shaded area.

The expected number of total deaths was 332,125 (95% bootstrapped CI, 331,405 to 332,846) in 2020, whereas the observed number of total deaths was 303,014. Therefore, the overall excess mortality was -29,112, and the observed deaths/expected deaths (O/E) ratio was 0.91. The overall mortality decreased by approximately 55 per 100,000 when normalized to the 2020 population in Korea. The excess mortality in 2020 was negative over the entire period except in week 5 (January 29 to February 4) (Figure 1). The cold-wave-adjusted estimate showed similar trend (Supplementary Material 4). The estimates calculated from vital statistics released by Statistics Korea showed a similar temporal pattern (Supplementary Material 5). The regional distribution of excess mortality is presented in Supplementary Materials 6 and 7. The scale of excess mortality was distributed in proportion to the population in each region. The O/E ratio was minimally associated with each region’s scale of the COVID-19 epidemic.

### Patterns of excess mortality by age group, sex, income, health coverage, and disability status

**Figure 2.**
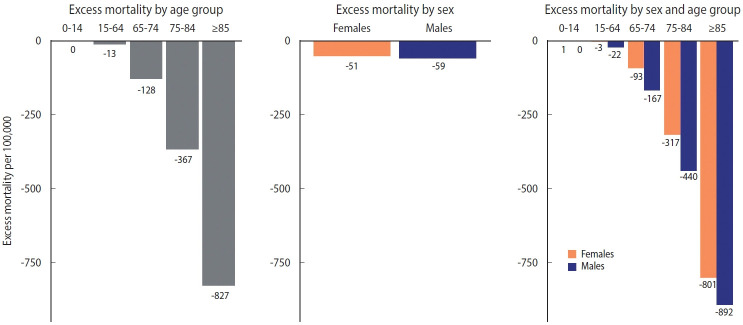
Excess mortality by sex and age group (per 100,000 persons).

**Figure 3.**
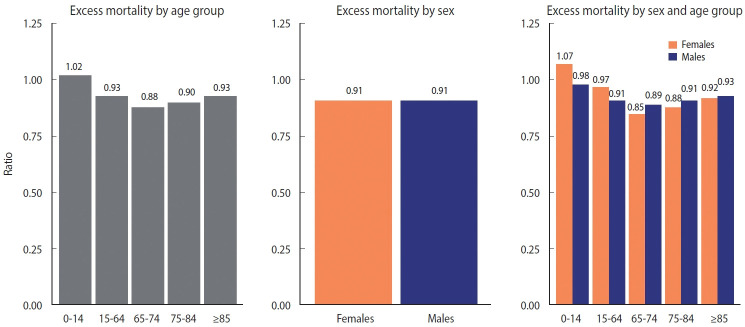
Excess mortality by sex and age group (ratio).

The distribution of excess mortality by age group and sex is shown in Figures 2 and 3. When the age was regrouped to intervals of 0-14, 15-64, 65-74, 75-84, and ≥ 85 in accordance with Islam et al. [10], the absolute magnitude of excess mortality increased linearly with age (meaning that fewer deaths than expected occurred in older age group). The difference by sex was the largest in the 75-84 age group and the smallest in the 0-14 age group. Males showed consistently larger absolute magnitudes of excess mortality than females within age groups (Figure 2). The relative indicators showed a somewhat different landscape. In relative form, excess mortality took on a U-shape. In the under-65 age group, the O/E ratio of females was larger than that of males; however, in the 65 and over age group, the ratio of males was larger than that of females (Figure 3).

**Figure 4.**
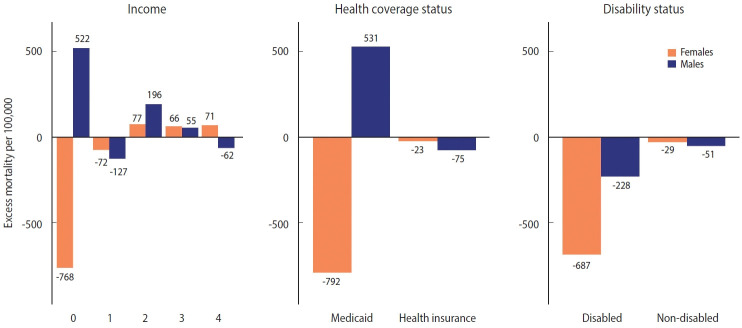
Excess mortality by income, health coverage, and disability status (per 100,000 persons).

**Figure 5.**
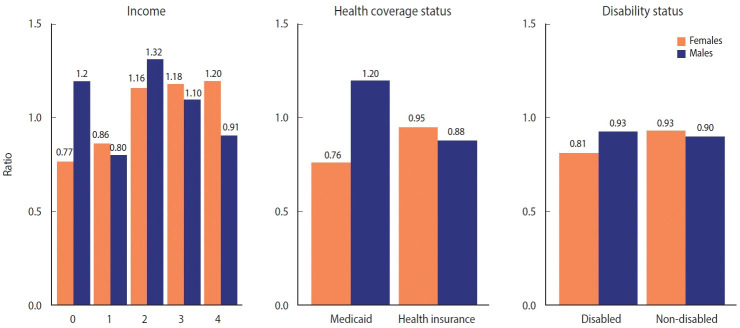
Excess mortality by income, health coverage, and disability status (ratio).

The patterns of excess mortality by income, health coverage, and disability status are shown in Figures 4 and 5. The income estimates showed notable variations. Contrary to expectations, positive excess mortality was observed in the higher-income group (groups 2 and 3), and negative values in the lower-income group (group 1). Males in groups 1 and 4 showed negative values, but males in group 0, eligible for Medical Aid, showed large positive excess mortality. Females in the lowest (0) and highest (4) income groups showed the opposite trend of excess mortality compared with males in the same income group. In contrast to males, females in the lowest income group showed the largest decrease in the death toll. Similarly, although both males and females with health insurance showed negative value, Medical Aid beneficiaries of different sexes showed the opposite result: increased mortality in males and decreased mortality in females. Disabled people showed much bigger absolute excess mortality than non-disabled people, but the ratio was similar. Disabled females showed fewer deaths than disabled males, but non-disabled females showed the opposite results. The bootstrapped 95% CIs were presented in Supplementary Material 8.

### Patterns of excess mortality by causes of death

**Figure 6.**
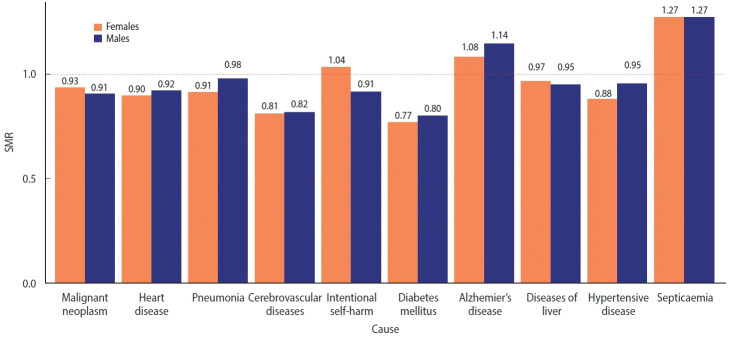
Standardized mortality rates (SMRs) of the top 10 causes of death in 2020.

The SMRs for the top 10 causes of death in 2020 were calculated (Figure 6). These 10 disease groups accounted for 67.9% of all deaths. Of the 10 disease groups, only Alzheimer’s disease and septicemia showed more deaths than expected, whereas the remaining causes of death decreased compared to the mortality in 2015-2019. The deaths from diabetes mellitus (0.79), cerebrovascular diseases (0.81), hypertensive diseases (0.90), and heart disease (0.91) were lower than expected. In addition, it is worth noting that deaths caused by intentional harm increased in females but decreased in males.

**Figure 7.**
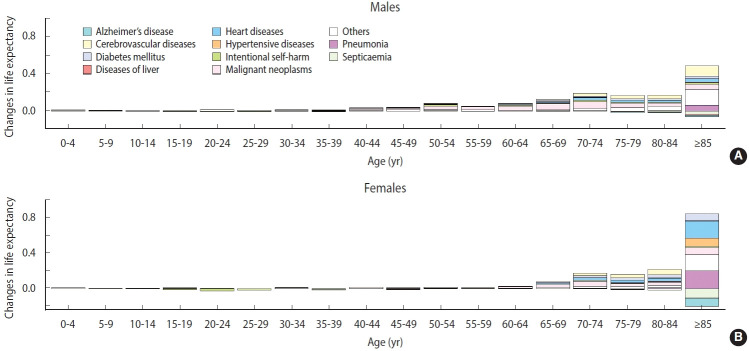
Comparison between 2020 and 2015-2019 using life expectancy decomposition.

The death certificate data were decomposed based on life expectancy (Figure 7). The increase in life expectancy due to negative excess mortality was mostly concentrated in the older population. The increase was the largest in those aged 85 years or more, and female’s life expectancy increased nearly 2-fold (0.88) compared to males in the same age group (0.42). The reduction in deaths from heart disease and pneumonia contributed significantly to the increased life expectancy, whereas deaths due to Alzheimer’s disease and septicemia increased. Unlike most population groups, young females (15-29 years) showed reduced life expectancy because of increased intentional self-harm.

## DISCUSSION

We estimated excess mortality using the NHID information collected between January 1 and December 31, 2020, in Korea. The estimated excess mortality in Korea was -29,124 (-55 per 100,000 persons). The O/E ratio was 0.91, which is lower than that in Australia (0.96) [26] or Japan (0.98) [27], despite the difficulty in undertaking a direct comparison due to methodological differences. Region had little effect on excess mortality in 2020. Negative excess mortality was observed in all sex-age groups, except for females in the 0-14 age group. The distribution by age group was U-shaped, and females who were younger than 65 years and males who were older than 65 years had higher excess mortality than their counterparts of the other sex in the same age group.

Depending on the health coverage status, negative excess mortality was identified, except for male Medical Aid beneficiaries, and negative values appeared in all groups according to disability status. When the SMR was calculated for the top 10 causes of death (accounting for 67.9% of all deaths), deaths from Alzheimer’s disease and septicemia increased, whereas deaths from other causes decreased. In particular, the decreases in deaths from diabetes mellitus (SMR, 0.79) and cerebrovascular disease (SMR, 0.81) were remarkable. Finally, we computed the life expectancy gap between 2015-2019 and 2020. The increase in life expectancy was mostly concentrated in the older adult population, whereas the life expectancy of young females (15-29 years) declined due to intentional self-harm.

The negative excess mortality observed in 2020 seems to be an anticipated result, as Korea is one of the countries that has implemented strict COVID-19 elimination strategies [7]. There were no significant regional variations in Korea, as the scale of the regional epidemic was negligible compared with that of other countries. The U-shaped distribution by age group can be explained by the influence of social distancing and the age-specific fatality rate of COVID-19. The 65-74 age group might have been located at the bottom of the U-shape because their biological risk is lower than that of the late elderly [28], and they might have experienced a lower financial impact from social distancing as they have already retired.

The results regarding the income group need to be interpreted with caution, as the mechanism for the insurance premium contribution of Korea’s National Health Insurance was reformed during the study period (the last major reform was in 2019). As pensioners continue to be included in the non-employer scheme, the negative excess mortality of income group 1 can be partially explained by a transfer of negative excess mortality that would have been observed in income groups 3 and 4 if there had been no changes in the insurance contribution scheme.

The larger absolute magnitude of the excess mortality rate relative to the O/E ratio in Medical Aid beneficiaries and the disabled group might be related to the smaller denominators than that of the general population. It is also noteworthy that, in the vulnerable population, male’s excess mortality showed different patterns from female’s, who enjoyed negative excess mortality. This pattern was also observed in the population aged 65 years and above, Medical Aid beneficiaries, and the disabled group. Although this variation needs in-depth investigation through follow-up studies, this finding might be attributed to differences between sexes in the coping capacity in disasters. This might be related to sex differences in self-care capacity and mutual aid, as the strict social distancing policy in the pandemic led to disruption of welfare programs. There is some evidence that females are more capable than males in self-care and reciprocal help in times of scarcity and continuing crisis [29,30]. However, the results from Medical Aid beneficiaries need further explanation in that males, but not females, showed positive excess mortality. Most of the excess mortality in Medical Aid beneficiaries occurred in the 15-64 age group. The number of males and females in this age group is similar, but the magnitude of excess mortality in males was nearly 3 times higher (Supplementary Material 9). In Korea, welfare recipients and middle-aged males (50-69) are known to have the highest risk of dying alone [31]. Because various social services provided by the state and the private sector have been suspended due to the COVID-19 pandemic, deaths of despair, including lonely deaths, may have occurred in male Medical Aid beneficiaries in this age group [32].

The increased Alzheimer’s disease and septicemia-related mortality could be explained by increased deaths in long-term facilities, which had difficulty transferring sick residents to acute hospitals during the pandemic. Delays in patient transfer might have contributed to the increase in non-specific causes of death, such as Alzheimer’s disease and septicemia. A relative decrease in deaths due to hypertensive disorders, heart disease, and cardiovascular diseases, which require additional diagnostic procedures at acute hospitals [33], might partially explain the increase in unknown causes of death. The “not elsewhere classified” group (R00-R99) increased from 9.5% in 2019 to 10.4% in 2020, with death codes for senility (R54) and other ill-defined and unspecified causes of mortality (R99) accounting for most of the increase. Some causes of death, like malignant neoplasms and diabetes mellitus, might have been accounted for as septicemia in hospitals, as usual medical services were interrupted by the pandemic [34-36]. However, when changes in absolute magnitude were calculated using the 2019 age-sex standardized mortality rate and the estimated SMR, this accounted for only 20% of the decrease in mortality, and further explanations for decreased mortality need to be explored. Lastly, the finding of increased fatalities from intentional harm in females is consistent with that of a previous study which used other data sources, and reflects the aggravation of female’s mental health during the pandemic itself [37] as well as during the economic recession caused by the pandemic [15,38].

This study identified a significantly larger negative excess mortality than previous studies, which estimated an excess mortality close to zero [12,13] and identified positive excess mortality [10,11]. Considering the granularity of the data used in each study, this constitutes an example of the coastline paradox, wherein the length of the coastline is measured longer when using smaller units. Therefore, the differences between the studies suggest the importance of data with proper granularity that are disaggregated to an appropriate level. Furthermore, the gap between international comparative studies is another issue. The estimates from comparative studies point in the opposite direction to those of domestic studies [10,11]. Unlike our study, which used the real observed mortality of 2020, a previous study analyzed the World Mortality Dataset [39], which used the predicted value of mortality structures for the death toll of 2020 from a previous period [10], and another study used a more aggregated dataset [11]. Countries that performed relatively well during the COVID-19 pandemic are not without faults. Thus, each country constantly referred to examples from other countries that have performed somewhat better in each phase. Excess mortality is a blunt indicator but has the potential to reveal the results of the overall performance of countries. This shows the importance of creating, sharing, and utilizing standardized mortality datasets to calculate this indicator and prepare for another future pandemic.

In line with international standards, this study computed excess mortality in Korea for the first time and showed that it is possible to monitor excess mortality using data from the NHIS, which is the single payer in Korea. However, this study had some limitations. First, this study only included data from 2020. Further analysis of data from subsequent periods will be essential as the COVID-19 pandemic continues. Second, the measurement of income quartiles was slightly unstable due to the reform in the system of calculating contributions. Korea has a single-payer model that ensures almost universal population coverage. The rules for determining the insurance contribution differ between wage earners and the self-employed group. The fairness of contributions for wage earners and those who are self-employed has long been controversial [40]. Therefore, the government changed the contribution scheme, and pensioners were transferred to the first quartile in 2019. However, we think that the income-related distribution of excess mortality still contains worthwhile results with careful interpretation. Third, the effects of temperature were not fully considered. Some studies utilized a separate model to explain the impact of temperature and undertook a 2-stage interrupted time series to assess excess mortality [27,41]. It is natural that the concept of excess mortality is usually used mainly to capture the effect of heat waves [42,43]. Although this study did not incorporate this methodology, we do not think that this constitutes a major obstacle to making internationally comparable estimates.

Our study adds essential evidence to the overall performance of Korea, one of the countries that adopted an elimination strategy during the early stages of the COVID-19 pandemic. As the negative excess mortality estimates show, the overall performance of Korea in 2020 was successful in protecting people’s lives. However, the inequalities according to various socioeconomic variables indicate that the fruits of intensive measures to control COVID-19 have not been distributed equitably. More efforts to properly evaluate the pandemic’s current and future health impacts are needed to mitigate them accordingly.

## SUPPLEMENTARY MATERIALS

Supplementary Material 1. Weekly COVID-19 cases and death counts

Supplementary Material 2 Definition of the week (Start date of the week)

Supplementary Material 3 General classification of causes of death (56 Items)

Supplementary Material 4 Weekly excess mortality in 2020 (cold wave adjusted)

Supplementary Material 5 Weekly excess mortality in 2020 (KOSIS data)

Supplementary Material 6 Regional excess mortality in 2020

Supplementary Material 7 Regional weekly excess mortality in 2020

Supplementary Material 8 Excess mortality by Age group, Sex, Income, Health coverage status, Disability status

Supplementary Material 9 Excess mortality profile of Medicaid beneficiaries
