# Supplementary figures and images for: Inequitable distribution of excess mortality during the COVID-19 pandemic in Korea, 2020
Source: Epidemiol Health. 2022 Sep 26;44:e2022081. doi: 10.4178/epih.e2022081 (PMC10089707; doi:10.4178/epih.e2022081)

**Supplementary Material 1. Weekly COVID-19 cases and death counts**

**
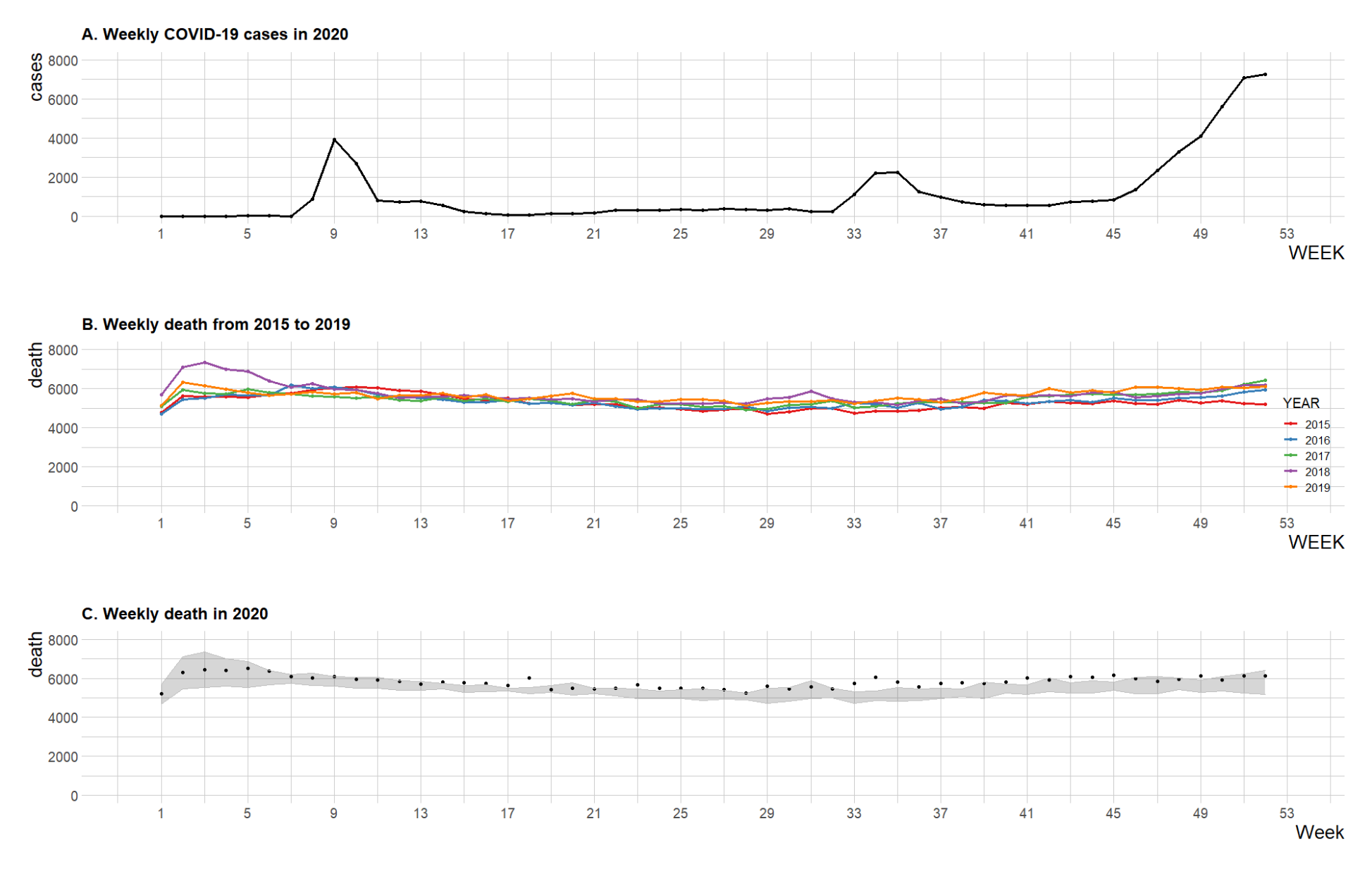
**

Supplement: Supplementary Material 1. — Weekly COVID-19 cases and death counts [file epih-44-e2022081-Supplementary-1.docx]

**Supplementary Material 4. Weekly excess mortality in 2020 (cold wave adjusted)**


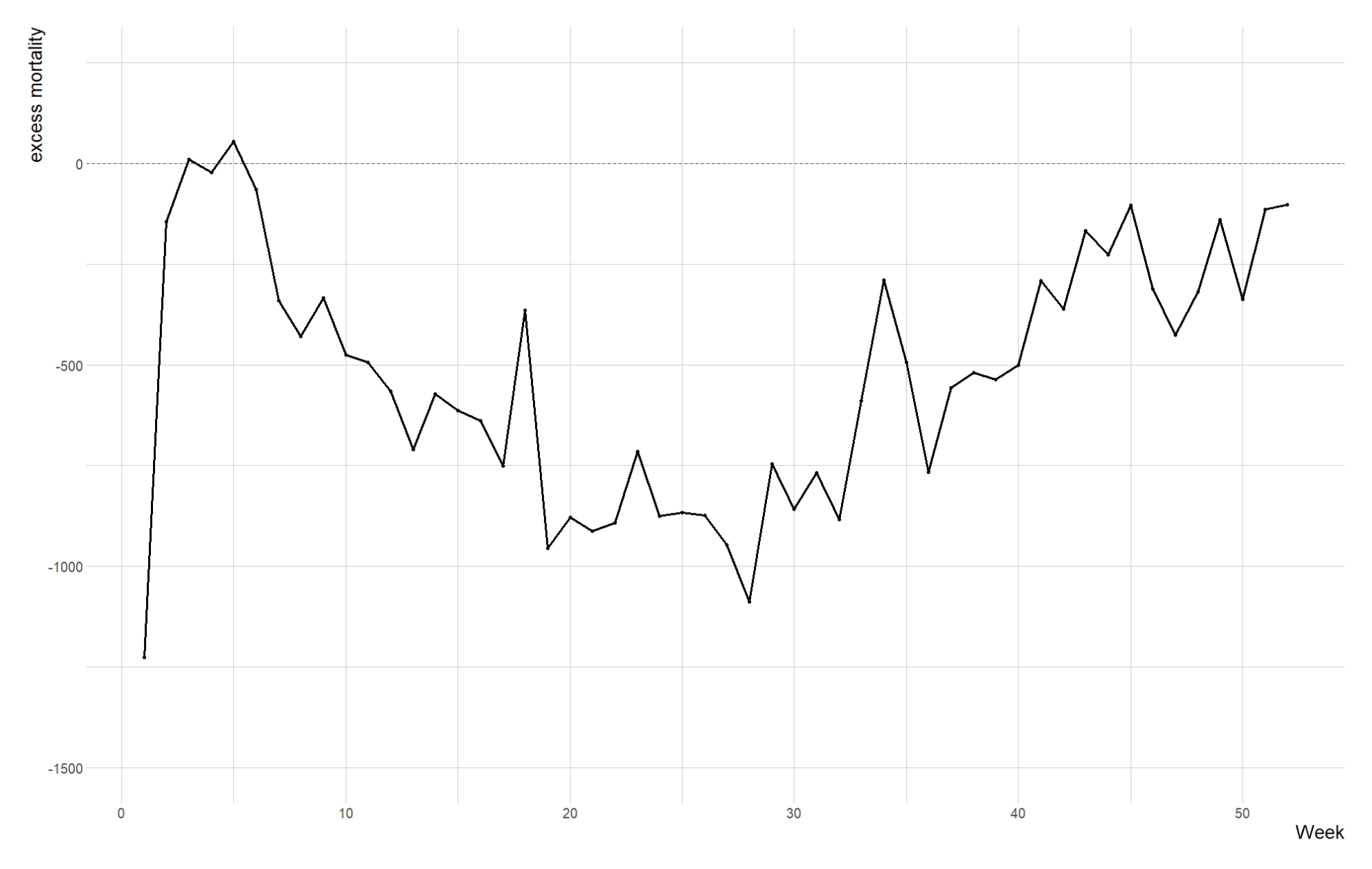

Supplement: Supplementary Material 4 — Weekly excess mortality in 2020 (cold wave adjusted) [file epih-44-e2022081-Supplementary-4.docx]

**Supplementary Material 5. Weekly excess mortality in 2020 (KOSIS data)**


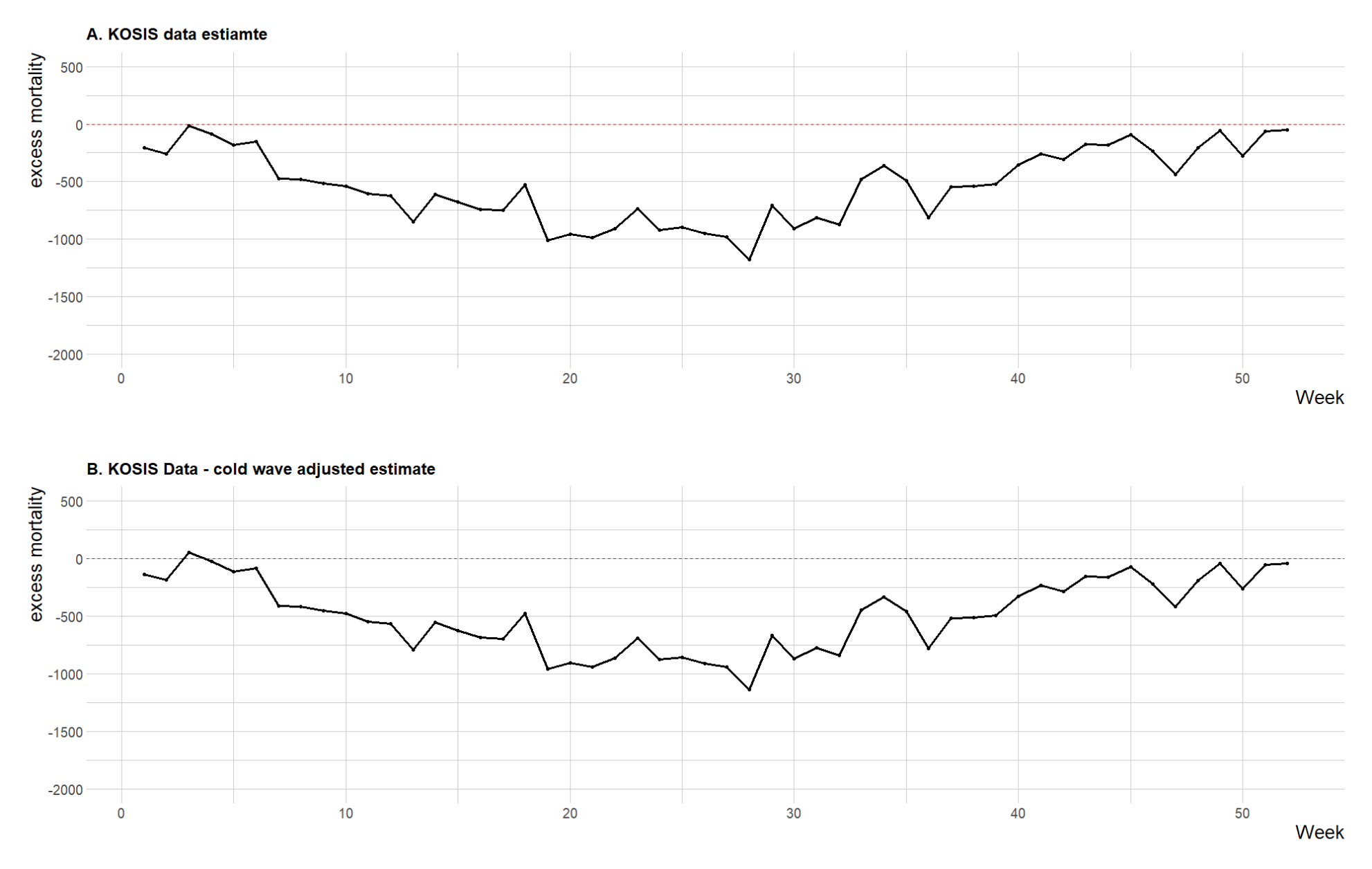

Supplement: Supplementary Material 5 — Weekly excess mortality in 2020 (KOSIS data) [file epih-44-e2022081-Supplementary-5.docx]

**Supplementary Material 7. Regional weekly excess mortality in 2020**

**
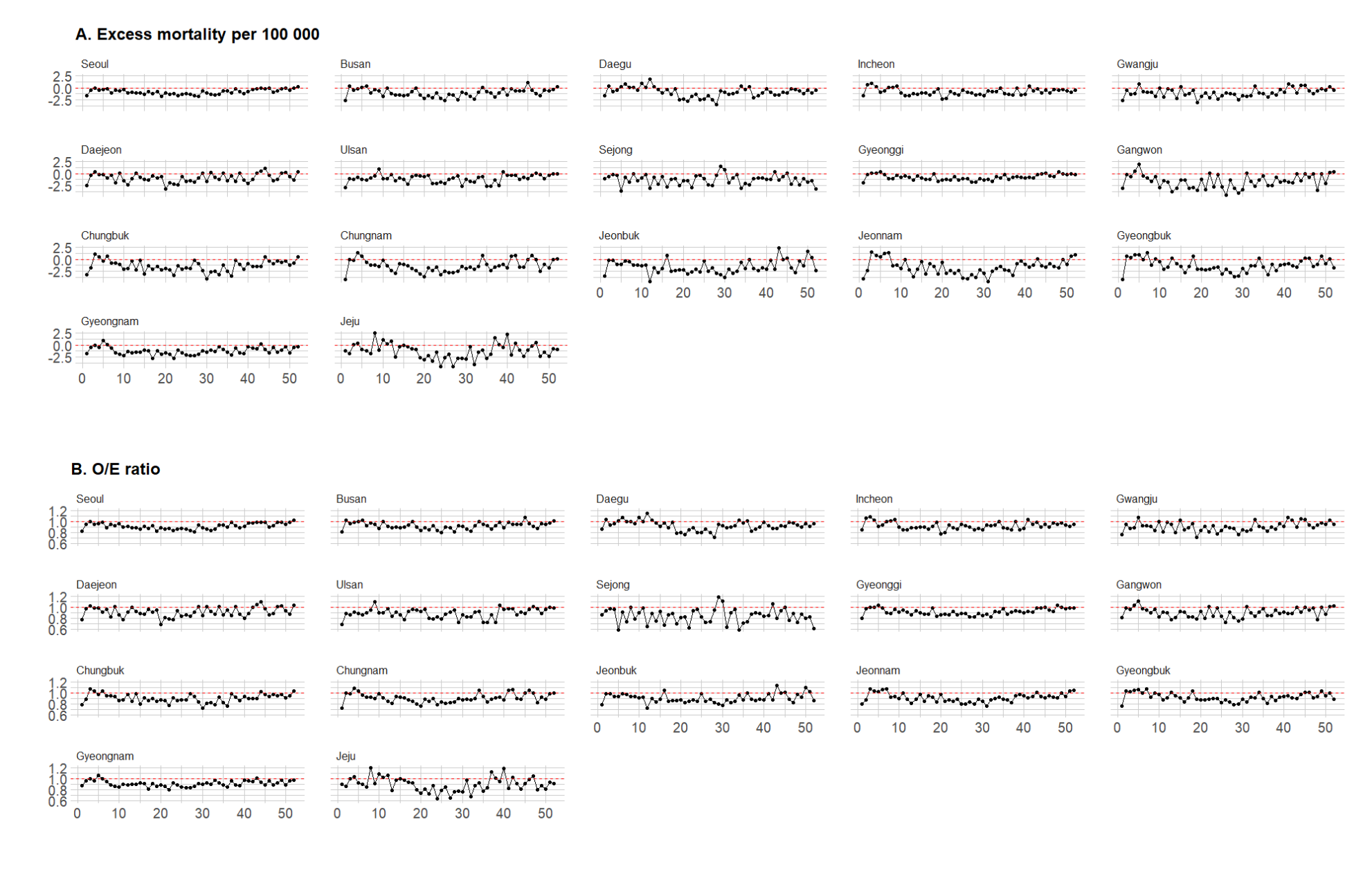
**

Supplement: Supplementary Material 7 — Regional weekly excess mortality in 2020 [file epih-44-e2022081-Supplementary-7.docx]
