# Supplementary material for: Inequitable distribution of excess mortality during the COVID-19 pandemic in Korea, 2020
Source: Epidemiol Health. 2022 Sep 26;44:e2022081. doi: 10.4178/epih.e2022081 (PMC10089707; doi:10.4178/epih.e2022081)
Supplement: Supplementary Material 2 — Definition of the week (Start date of the week) [file epih-44-e2022081-Supplementary-2.docx]

**Supplementary Material 2. Definition of the week (Start date of the week)**

| **Week** | **2015** | **2016** | **2017** | **2018** | **2019** | **2020** |
| --- | --- | --- | --- | --- | --- | --- |
| **1** | 20150101 | 20160101 | 20170101 | 20180101 | 20190101 | 20200101 |
| **2** | 20150108 | 20160108 | 20170108 | 20180108 | 20190108 | 20200108 |
| **3** | 20150115 | 20160115 | 20170115 | 20180115 | 20190115 | 20200115 |
| **4** | 20150122 | 20160122 | 20170122 | 20180122 | 20190122 | 20200122 |
| **5** | 20150129 | 20160129 | 20170129 | 20180129 | 20190129 | 20200129 |
| **6** | 20150205 | 20160205 | 20170205 | 20180205 | 20190205 | 20200205 |
| **7** | 20150212 | 20160212 | 20170212 | 20180212 | 20190212 | 20200212 |
| **8** | 20150219 | 20160219 | 20170219 | 20180219 | 20190219 | 20200219 |
| **9** | 20150226 | 20160226 | 20170226 | 20180226 | 20190226 | 20200226 |
| **10** | 20150305 | 20160304 | 20170305 | 20180305 | 20190305 | 20200304 |
| **11** | 20150312 | 20160311 | 20170312 | 20180312 | 20190312 | 20200311 |
| **12** | 20150319 | 20160318 | 20170319 | 20180319 | 20190319 | 20200318 |
| **13** | 20150326 | 20160325 | 20170326 | 20180326 | 20190326 | 20200325 |
| **14** | 20150402 | 20160401 | 20170402 | 20180402 | 20190402 | 20200401 |
| **15** | 20150409 | 20160408 | 20170409 | 20180409 | 20190409 | 20200408 |
| **16** | 20150416 | 20160415 | 20170416 | 20180416 | 20190416 | 20200415 |
| **17** | 20150423 | 20160422 | 20170423 | 20180423 | 20190423 | 20200422 |
| **18** | 20150430 | 20160429 | 20170430 | 20180430 | 20190430 | 20200429 |
| **19** | 20150507 | 20160506 | 20170507 | 20180507 | 20190507 | 20200506 |
| **20** | 20150514 | 20160513 | 20170514 | 20180514 | 20190514 | 20200513 |
| **21** | 20150521 | 20160520 | 20170521 | 20180521 | 20190521 | 20200520 |
| **22** | 20150528 | 20160527 | 20170528 | 20180528 | 20190528 | 20200527 |
| **23** | 20150604 | 20160603 | 20170604 | 20180604 | 20190604 | 20200603 |
| **24** | 20150611 | 20160610 | 20170611 | 20180611 | 20190611 | 20200610 |
| **25** | 20150618 | 20160617 | 20170618 | 20180618 | 20190618 | 20200617 |
| **26** | 20150625 | 20160624 | 20170625 | 20180625 | 20190625 | 20200624 |
| **27** | 20150702 | 20160701 | 20170702 | 20180702 | 20190702 | 20200701 |
| **28** | 20150709 | 20160708 | 20170709 | 20180709 | 20190709 | 20200708 |
| **29** | 20150716 | 20160715 | 20170716 | 20180716 | 20190716 | 20200715 |
| **30** | 20150723 | 20160722 | 20170723 | 20180723 | 20190723 | 20200722 |
| **31** | 20150730 | 20160729 | 20170730 | 20180730 | 20190730 | 20200729 |
| **32** | 20150806 | 20160805 | 20170806 | 20180806 | 20190806 | 20200805 |
| **33** | 20150813 | 20160812 | 20170813 | 20180813 | 20190813 | 20200812 |
| **34** | 20150820 | 20160819 | 20170820 | 20180820 | 20190820 | 20200819 |
| **35** | 20150827 | 20160826 | 20170827 | 20180827 | 20190827 | 20200826 |
| **36** | 20150903 | 20160902 | 20170903 | 20180903 | 20190903 | 20200902 |
| **37** | 20150910 | 20160909 | 20170910 | 20180910 | 20190910 | 20200909 |
| **38** | 20150917 | 20160916 | 20170917 | 20180917 | 20190917 | 20200916 |
| **39** | 20150924 | 20160923 | 20170924 | 20180924 | 20190924 | 20200923 |
| **40** | 20151001 | 20160930 | 20171001 | 20181001 | 20191001 | 20200930 |
| **41** | 20151008 | 20161007 | 20171008 | 20181008 | 20191008 | 20201007 |
| **42** | 20151015 | 20161014 | 20171015 | 20181015 | 20191015 | 20201014 |
| **43** | 20151022 | 20161021 | 20171022 | 20181022 | 20191022 | 20201021 |
| **44** | 20151029 | 20161028 | 20171029 | 20181029 | 20191029 | 20201028 |
| **45** | 20151105 | 20161104 | 20171105 | 20181105 | 20191105 | 20201104 |
| **46** | 20151112 | 20161111 | 20171112 | 20181112 | 20191112 | 20201111 |
| **47** | 20151119 | 20161118 | 20171119 | 20181119 | 20191119 | 20201118 |
| **48** | 20151126 | 20161125 | 20171126 | 20181126 | 20191126 | 20201125 |
| **49** | 20151203 | 20161202 | 20171203 | 20181203 | 20191203 | 20201202 |
| **50** | 20151210 | 20161209 | 20171210 | 20181210 | 20191210 | 20201209 |
| **51** | 20151217 | 20161216 | 20171217 | 20181217 | 20191217 | 20201216 |
| **52** | 20151224 | 20161223 | 20171224 | 20181224 | 20191224 | 20201223 |
| **53** | 20151231 | **20161230** | 20171231 | 20181231 | 20191231 | **20201230** |
