# Supplementary material for: Inequitable distribution of excess mortality during the COVID-19 pandemic in Korea, 2020
Source: Epidemiol Health. 2022 Sep 26;44:e2022081. doi: 10.4178/epih.e2022081 (PMC10089707; doi:10.4178/epih.e2022081)
Supplement: Supplementary Material 3 — General classification of causes of death (56 Items) [file epih-44-e2022081-Supplementary-3.docx]

**Supplementary Material 3. General classification of causes of death (56 Items)**

| **Statistics Korea** | **Cause of death** | **KCD code** |
| --- | --- | --- |
| 001 | Cholera | A00 |
| 002 | Other gastroenteritis and colitis of infectious and unspecified origin | A09 |
| 003 | Other intestinal infectious diseases | A01–A08 |
| 004 | Respiratory tuberculosis | A15, A16 |
| 005 | Other tuberculosis | A17–A19 |
| 006 | Plague | A20 |
| 007 | Tetanus | A33–A35 |
| 008 | Diphtheria | A36 |
| 009 | Whooping cough | A37 |
| 010 | Meningococcal infection | A39 |
| 011 | Septicemia | A40–A41 |
| 012 | Infections with a predominantly sexual mode of transmission | A50–A64 |
| 013 | Acute poliomyelitis | A80 |
| 014 | Rabies | A82 |
| 015 | Yellow fever | A95 |
| 016 | Other arthropod-borne viral fevers and viral hemorrhagic fevers | A90–A94, A96–A99 |
| 017 | Measles | B05 |
| 018 | Viral hepatitis | B15–B19 |
| 019 | Human immunodeficiency virus[HIV] disease | B20–B24 |
| 020 | Malaria | B50–B54 |
| 021 | Leishmaniasis | B55 |
| 022 | Trypanosomiasis | B56, B57 |
| 023 | Schistosomiasis | B65 |
| 024 | Remainder of infectious and parasitic diseases | A21–A32, A38, A42–A49, A65–A79, A81, A83–A89, B00–B04, B06–B09, B25–B49, B58–B64, B66–B94, B99 |
| 025 | Malignant neoplasms | C00–C97 |
| 026 | Anemias | D50–D64 |
| 027 | Diabetes mellitus | E10–E14 |
| 028 | Malnutrition | E40–E46 |
| 029 | Mental and behavioral disorders due to psychoactive substance use | F10–F19 |
| 030 | Meningitis | G00, G03 |
| 031 | Alzheimer's disease | G30 |
| 032 | Acute rheumatic fever and chronic rheumatic heart diseases | I00–I09 |
| 033 | Hypertensive diseases | I10–I13 |
| 034 | Heart diseases | I20–I51 |
| 035 | Cerebrovascular diseases | I60–I69 |
| 036 | Atherosclerosis | I70 |
| 037 | Influenza | J09–J11 |
| 038 | Pneumonia | J12-J18 |
| 039 | Other acute lower respiratory infections | J20–J22, U04 |
| 040 | Chronic lower respiratory diseases | J40–J47 |
| 041 | Gastric and duodenal ulcers | K25–K27 |
| 042 | Diseases of the liver | K70–K76 |
| 043 | Glomerular and renal tubulointerstitial diseases | N00–N15 |
| 044 | Pregnancy with abortive outcome | O00–O07 |
| 045 | Other direct obstetric deaths | O10–O92 |
| 046 | Indirect obstetric deaths | O98, O99 |
| 047 | Certain conditions originating in the perinatal period | P00–P96 |
| 048 | Congenital malformations, deformations, and chromosomal abnormalities | Q00–Q99 |
| 049 | Sudden infant death syndrome | R95 |
| 050 | Transport accidents | V01–V99 |
| 051 | Falls | W00–W19 |
| 052 | Accidental drowning and submersion | W65–W74 |
| 053 | Exposure to smoke, fire, and flames | X00–X09 |
| 054 | Accidental poisoning by, and exposure to, noxious substances | X40–X49 |
| 055 | Intentional self-harm | X60–X84 |
| 056 | Assault | X85–Y09 |
