# Supplementary material for: Inequitable distribution of excess mortality during the COVID-19 pandemic in Korea, 2020
Source: Epidemiol Health. 2022 Sep 26;44:e2022081. doi: 10.4178/epih.e2022081 (PMC10089707; doi:10.4178/epih.e2022081)
Supplement: Supplementary Material 6 — Regional excess mortality in 2020 [file epih-44-e2022081-Supplementary-6.docx]

**Supplementary Material 6. Regional excess mortality in 2020**

| **Region** | **Deaths** | | | | | |
| --- | --- | --- | --- | --- | --- | --- |
|  | **Observed** | **Expected** | **Excess mortality** | **Rate^a^** | **O/E ratio** | **COVID-19^b^** |
| **Total** | 303,014 | 332,125 (331,405-332,846) | −29,112 (−29,832 to −28,391) | −54.9 | 0.91 | 915 |
| **Seoul** | 45,733 | 50,073 (49,774-50,373) | -4,340 (-4,640 to -4,041) | -43.7 | 0.91 | 182 |
| **Busan** | 22,598 | 24,542 (24,364-24,720) | -1,944 (-2,122 to -1,766) | -56.7 | 0.92 | 50 |
| **Daegu** | 14,190 | 15,400 (15,266-15,533) | -1,210 (-1,343 to -1,076) | -49.3 | 0.92 | 201 |
| **Incheon** | 15,511 | 16,829 (16,694-16,963) | -1,318 (-1,452 to -1,183) | -43.5 | 0.92 | 29 |
| **Gwangju** | 7,497 | 8,295 (8,205-8,386) | -798 (-889 to -708) | -54.2 | 0.9 | 6 |
| **Daejeon** | 7,531 | 8,221 (8,133-8,309) | -690 (-778 to -602) | -46.4 | 0.92 | 7 |
| **Ulsan** | 5,149 | 5,834 (5,756-5,913) | -685 (-764 to -607) | -58.5 | 0.88 | 26 |
| **Sejong** | 1,265 | 1,500 (1,465-1,536) | -235 (-271 to -200) | -67.7 | 0.84 | 1 |
| **Gyeong-gi** | 62,515 | 68,201 (67,830-68,572) | -5,686 (-6,057 to -5,315) | -41.4 | 0.92 | 271 |
| **Gang-won** | 12,088 | 13,415 (13,285-13,546) | -1,327 (-1,458 to -1,197) | -85.5 | 0.9 | 13 |
| **Chung-buk** | 11,527 | 12,788 (12,666-12,909) | -1,261 (-1,382 to -1,139) | -76.7 | 0.9 | 24 |
| **Chung-nam** | 15,841 | 17,515 (17,370-17,660) | -1,674 (-1,819 to -1,529) | -76.1 | 0.9 | 23 |
| **Jeon-buk** | 14,557 | 16,087 (15,947-16,227) | -1,530 (-1,670 to -1,390) | -83.2 | 0.9 | 11 |
| **Jeon-nam** | 17,588 | 19,256 (19,097-19,416) | -1,668 (-1,828 to -1,509) | -88 | 0.91 | 5 |
| **Gyeong-buk** | 22,727 | 24,647 (24,464-24,830) | -1,920 (-2,103 to -1,737) | -70.9 | 0.92 | 62 |
| **Gyeong-nam** | 22,767 | 25,114 (24,921-25,307) | -2,347 (-2,540 to -2,154) | -68.4 | 0.91 | 4 |
| **Jeju** | 3,930 | 4,409 (4,342-4,476) | -479 (-546 to -412) | -69.4 | 0.89 | 0 |

COVID-19, coronavirus disease; O/E, observed death/expected death ratio.

^a^ Excess mortality per 100,000 people in each region.

^b^ COVID-19 mortality data were retrieved from the Korea Center for Disease Control and Prevention Agency; two deaths classified as quarantine (overseas entrant) were excluded.
