# Supplementary material for: Inequitable distribution of excess mortality during the COVID-19 pandemic in Korea, 2020
Source: Epidemiol Health. 2022 Sep 26;44:e2022081. doi: 10.4178/epih.e2022081 (PMC10089707; doi:10.4178/epih.e2022081)
Supplement: Supplementary Material 8 — Excess mortality by Age group, Sex, Income, Health coverage status, Disability status [file epih-44-e2022081-Supplementary-8.docx]

**Supplementary Material 8. Excess mortality by Age group, Sex, Income, Health coverage status, Disability status**

|  | | **Excess mortality** | | | **O/E ratio** | | |
| --- | --- | --- | --- | --- | --- | --- | --- |
|  |  | **Total** | **Female** | **Male** | **Total** | **Female** | **Male** |
| **Age group** | 0-14 | 16  (-5 to 37) | 28 (15-41) | -12  (-29 to 4) | 1.02  (0.99-1.04) | 1.07  (1.04-1.11) | 0.98  (0.95-1.01) |
|  | 15-64 | -4,807  (-5,502 to -4,562) | -576  (-696 to -455) | -4,231  (-4,445 to -4,017) | 0.93  (0.93-0.93) | 0.97  (0.96-0.98) | 0.91  (0.91-0.92) |
|  | 65-74 | -6,323  (-6,566 to -6,080) | -2,397  (-2,524 to -2,269) | -3,926  (-4,129 to -3,724) | 0.88  (0.88-0.89) | 0.85  (0.85-0.86) | 0.89  (0.89-0.90) |
|  | 75-84 | -10,402  (-10,750 to -10,054) | -5,292  (-5,541 to -5,044) | -5,110  (-5,716 to -4,832) | 0.90  (0.90-0.90) | 0.88  (0.88-0.89) | 0.91  (0.91-0.91) |
|  | 85+ | -7,596  (-8,092 to -7,101) | -5,274  (-5,716 to -4,82) | -2,322  (-2,550 to -2,095) | 0.93  (0.92-0.93) | 0.92  (0.92-0.93) | 0.93  (0.93-0.94) |
| **Income** | 0 (Medicaid) | -2,568  (-2,869 to -2,266) | -6,153  (-6,344 to -5,962) | 3,585  (3,454-3,717) | 0.94  (0.94-0.95) | 0.77  (0.76-0.77) | 1.20  (1.19-1.20) |
|  | 1 | -39,850  (-40,800 to -38,900) | -14,404  (-14,920 to -13,888) | -25,446  (-26,001 to -24,891) | 0.83  (0.83-0.83) | 0.86  (0.86-0.87) | 0.80  (0.80-0.81) |
|  | 2 | 4,292  (4,215-4,368) | 1,179  (1,140-1,219) | 3,112  (3,066-3,158) | 1.25  (1.24-1.25) | 1.16  (1.15-1.17) | 1.32  (1.31-1.32) |
|  | 3 | 2,287  (2,212-2,362) | 1,248  (1,212-1,2840 | 1,039  (992-1,086) | 1.13  (1.13-1.14) | 1.18  (1.17-1.19) | 1.10  (1.09-1.10) |
|  | 4 | 201  (98-304) | 1,644  (1,599-1,689) | -1,443  (-1,512 to -1,374) | 1.01  (1.00-1.01) | 1.20  (1.19-1.21) | 0.91  (0.90-0.91) |
| **Health coverage** | Medicaid | -2,704  (-3,023 to -2,704) | -6,350  (-6,556 to -6,143) | 3,645  (3,505-3,785) | 0.94  (0.93-0.95) | 0.76  (0.76-0.77) | 1.20  (1.19-1.21) |
|  | Health Insurance | -25,341  (-26,102 to -24,581) | -6,011  (-6,533 to -5,470) | -19,330  (-19,799 to -18,861) | 0.91  (0.91-0.91) | 0.95  (0.95-0.96) | 0.88  (0.88-0.88) |
| **Disability** | Disabled | -11,512  (-11,948 to -11,075) | -7,902  (-8,162 to -7,641) | -3,610  (-3,869 to -3,351) | 0.88  (0.87-0.88) | 0.81  (0.81-0.82) | 0.93  (0.92-0.93) |
|  | Non-disabled | -20,011  (-20,689 to -19,332) | -7,342  (-7,821 to -6,863) | -12,669  (-13,080 to -12,258) | 0.92  (0.91-0.92) | 0.93  (0.93-0.93) | 0.90  (0.90-0.91) |

O/E, observed death/expected death ratio.
