# Supplementary material for: Inequitable distribution of excess mortality during the COVID-19 pandemic in Korea, 2020
Source: Epidemiol Health. 2022 Sep 26;44:e2022081. doi: 10.4178/epih.e2022081 (PMC10089707; doi:10.4178/epih.e2022081)
Supplement: Supplementary Material 9 — Excess mortality profile of Medicaid beneficiaries [file epih-44-e2022081-Supplementary-9.docx]

**Supplementary Material 9. Excess mortality profile of Medicaid beneficiaries**

|  | | **Population** | **Excess mortality** | |
| --- | --- | --- | --- | --- |
|  |  |  | **Total** | **Per 100,000** |
| **Male** | **0-14** | 53,632 | 23 | 43 |
|  | **15-64** | 425,900 | 5,827 | 1,368 |
|  | **65-74** | 120,192 | 1,408 | 1,171 |
|  | **75-84** | 68,903 | -1,592 | -2,311 |
|  | **85+** | 18,507 | -2,016 | -10,893 |
| **Female** | **0-14** | 51,952 | 14 | 26 |
|  | **15-64** | 386,409 | 2,155 | 558 |
|  | **65-74** | 133,056 | 663 | 449 |
|  | **75-84** | 145,614 | -2,051 | -1,409 |
|  | **85+** | 83,664 | -7,122 | -8,513 |
